# Medicinal and Environmental Indicator Species of *Utricularia* from Montane Forest of Peninsular Malaysia

**DOI:** 10.1100/2012/234820

**Published:** 2012-04-30

**Authors:** Noorma Wati Haron, Ming Yee Chew

**Affiliations:** ^1^Institute of Biological Sciences, Faculty of Science, University of Malaya, 50603 Kuala Lumpur, Malaysia; ^2^Kepong Herbarium, Forest Research Institute Malaysia, 52109 Kepong, Selangor, Malaysia

## Abstract

The carnivorous *Utricularia* (Lentibulariaceae) is a small herb of multifarious wet habitats worldwide. Eleven of the 14 Peninsular Malaysian species range into the mountains. Distribution, disturbance adaptability and collection frequency were used to formulate their commonness category. Common (*U. aurea*, *U. bifida*, and *U. minutissima*) and fairly common (*U. gibba* and *U. uliginosa*) species are mostly lowland plants that ascend to open montane microhabitats, while the fairly common (*U. striatula*), narrow-range (*U. caerulea* pink form and *U. involvens*), rare (*U. furcellata* and *U. scandens*), and endemic (*U. vitellina*) species are restricted to mountainous sites. Common species that colonise dystrophic to oligotrophic man-made sites in late succession could serve as predictors for general health and recovery of wet habitats. Rarer species are often locally abundant, their niches situated around pristine forest edges. When in decline, they indicate the beginning of problems affecting the forest. *Utricularia* is reportedly nutritious, mildly astringent, and diuretic. Preadapted to nutrient-poor, waterlogged soils, *U. bifida* is suitable as an alternative for small-scale herb cultivation on low pH, wet poor soils usually deemed not suitable for any crops.

## 1. Introduction

Almost half of the approximately 500 carnivorous angiosperm species are from the genus *Utricularia* L., the bladderworts (family Lentibulariaceae), which has a cosmopolitan distribution in multifarious wet habitats worldwide [1]. Peninsular Malaysia is home to 14 *Utricularia *species, 11 (Table 1) of which ranges extend into the mountainous habitats while five are almost restricted to mountains. The body plan of *Utricularia* is peculiar, plastic, and unique among flowering plants. It is capable of changing its resources investment to match varying water chemistry, irradiance level, and prey availability [2, 3].

The cost-benefit model by Givnish et al. [4] showed that carnivorous plants generally prefer sunny, moist, low-nutrient habitats with low pH (3–7). Many aspects of the ecology and carnivorous habit of *Utricularia *have been researched previously [5–7]. One third of Peninsular Malaysian *Utricularia* are habitat specialists, that is, requiring strict edaphic conditions and niches to survive, another third are habitat generalists that are found in many sites and are suited to live in many types of wet microhabitats but are rarely found in heavily disturbed sites, while three species are common pioneers of open and wet habitats [8]. This study investigated the montane microhabitat characteristics of *Utricularia *in Peninsular Malaysia, documenting their commonness, and establishing their potential as environmental indicators and as medicinal species.

## 2. Materials and Methods

Baseline distribution information from herbaria specimens was tabulated followed by field surveys to record microhabitat physical and biotic details. Herbarium specimens examined and vouchers collected during field surveys are listed in Table 2. In Peninsular Malaysia, the montane forest formation includes both the lower ((600–)800–1,500 m) and upper (>1,500 m) montane forest [10]. Here, steeply hilly riverine sites above 300 m are also included for discussion. Montane diversity hotspots, localities with rare species, and potential sites with suitable habitats for *Utricularia* were targeted. An empirical commonness category was formulated based on the distribution of each species in Peninsular Malaysia, its adaptability to disturbance and collection frequency that indicates rarity (Table 3). The two subcategories of common species include those distributed throughout Peninsular Malaysia; on the opposite scale narrowly ranging and rare species are only found in a few localities.

## 3. Results and Discussion

**Figure 1:**
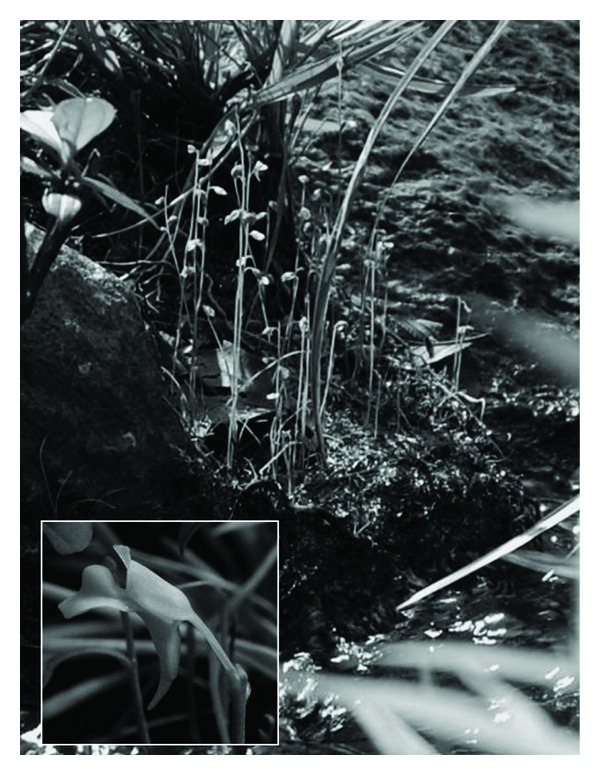
*Utricularia bifida*—the most common terrestrial species often found along waysides.

**Figure 2:**
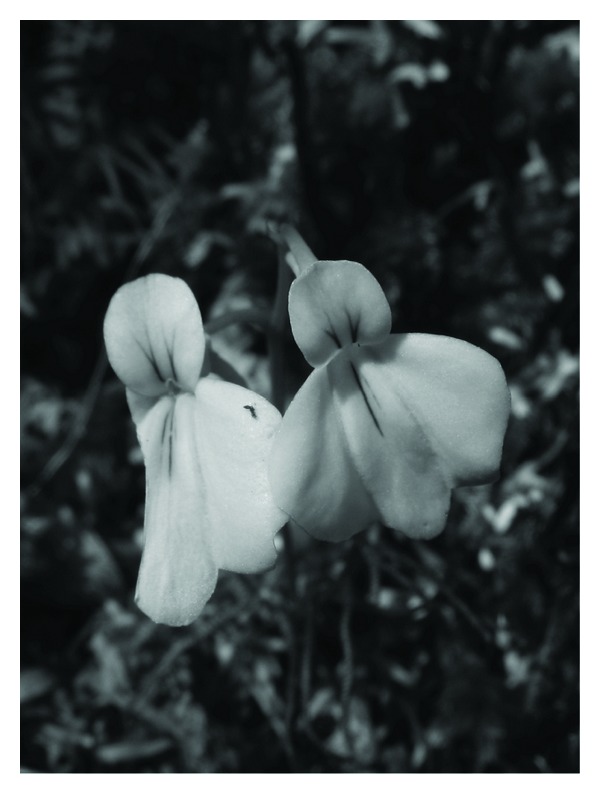
*Utricularia vitellina—*the endemic montane species of Peninsular Malaysia.

*Utricularia *species occupy a variety of microhabitats in mountainous areas (Table 4). *Utricularia aurea*, *U*.* bifida *(Figure 1), and *U*.* minutissima *are the three most common species throughout Peninsular Malaysia, while *U*.* gibba*,* U*.* striatula*, and *U*. *uliginosa *are from the fairly common category, found mainly in natural sites throughout Peninsular Malaysia. Except for *U*.* striatula*, they are mostly lowland plants, ascending the mountain along streams, heath, and man-made, ephemeral wetlands. *Utricularia striatula* is essentially a montane species but descends to the lowland along streams and is restricted to perpetually wet and humid microhabitats. *U*.* caerulea *(pink form) and *U*.* involvens *are narrow-range taxa. There are three rare species, *U*.* furcellata *and *U*.* scandens *are each found in a single locality, while *U*.* vitellina *(Figure 2) is found in two localities. All these narrow-range and rare taxa are restricted to mountainous sites.

**Figure 3:**
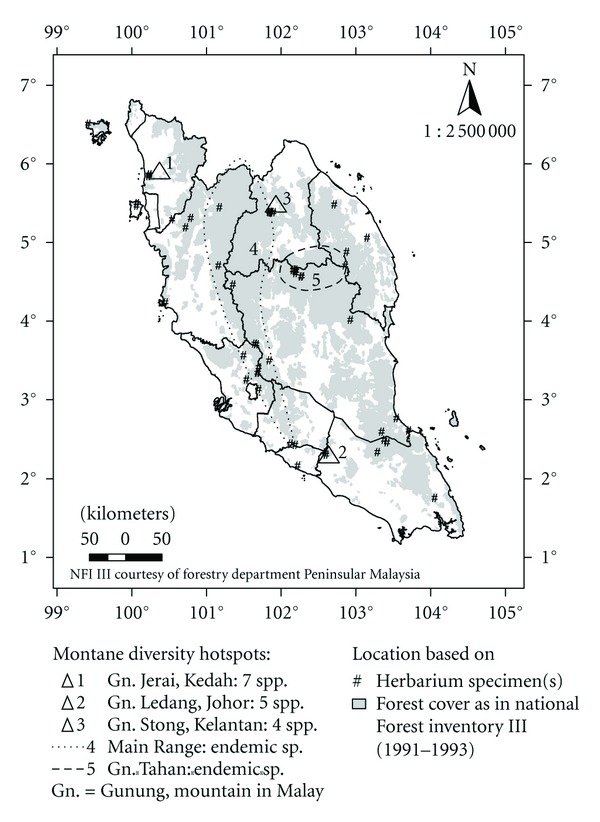
Distribution of *Utricularia *in mountainous habitats in Peninsular Malaysia (≥300 m a.s.l.).

The distribution pattern of the genus (Figure 3) illustrates that diversity hotspots are centred in small isolated mountain massifs. Gunung Jerai, Kedah, topped the list with seven species, followed by Gunung Ledang, Johor with five species. The endemic *U*.* vitellina *is restricted to one type of microhabitat, that is, peaty and mossy stream banks within the lower and upper montane forest, on the two highest peaks in Peninsular Malaysia, namely, Gunung Korbu and Gunung Tahan. The rare *U*.* furcellata *is confined to a small heath-like sandy patch on Gunung Ayam, Kelantan, while *U*.* scandens* to the stony heath on Gunung Mering, Johor. Mountains with large, open, montane heath, or swamps harvest extremely large *Utricularia *populations; for example, the *padang* (rock field) of Gunung Tahan, Pahang, and the *Sphagnum* bog of Gunung Stong, Kelantan.

### 3.1. Potential as Environmental Indicator

The presence of common pioneer species (*Utricularia aurea *and* U*.* bifida*) indicates past disturbance in a habitat, such as that demonstrated in a paleonological study, with the first appearance of *U*. *aurea *pollen coinciding with the arrival of aborigines in Tasik Bera, Pahang [11]. The common or fairly common species may colonise pond edges and shallowly inundated patches in well-established gardens, constructed wetlands, or waysides in late succession. They are, however, absent from heavily worked agricultural land or sites that are observably affected by severe chemical or organic waste runoffs and siltation. A few species are cultivated by local as well as international enthusiasts as curiosity plant, hence their requirements for dystrophic to oligotrophic conditions have been documented. Many mountainous areas in Peninsular Malaysia are being developed as recreational destinations with extensive landscaping, while large stretches are sought after by the agricultural sector to cultivate cash crops that need a more temperate environment to grow. The presence of common *Utricularia *species in the suburban waysides can therefore be used as a rough prediction for water trophicity, succession stages, and the general health of the regenerating secondary patches.

Narrow-range and rare species occupy wet montane habitats with acidic soil (pH range 3.5–6), either along stream-beds (*U*. *caerulea*,* U. involvens*, and *U. vitellina*) or heath (*U*.* furcellata *and *U. scandens*). Under pristine conditions, these species are often locally abundant. Despite that, rare species historically recorded from sites that were later affected by heavy hiking traffic, recreation amenity development, drying-up effect from loss of vegetation in the surrounding area, and flash-floods often did not survive this microhabitat loss. They are therefore indicators of environmental degradation. Parks and protected forest areas are continuously being used by the public for recreational and educational purposes. In order to achieve their species conservation roles while allowing for controlled use within their carrying capacities, these areas need to be monitored periodically for their general health. As *Utricularia *occupies niches that are nestled around the edges of the forest but rarely within it, their decline could herald the beginning of major problems that would affect the rest of the forest communities if left unchecked.

### 3.2. Potential as Medicinal Crop

*Utricularia* is recorded to be edible and high in nutrients. Some species used as folk remedies are mildly astringent and diuretic. *U*.* caerulea* is used to dress wounds while *U*.* bifida *is used to treat urinary diseases. Although yet to be widely researched, the medicinal potential of this species-rich genus is immense. In Peninsular Malaysia, *U*.* bifida *and *U*.* minutissima *are pioneers of open disturbed wetland and are often locally abundant, although *U*.* caerulea *is increasingly rare. *U*.* bifida *is highly suited for acidic damp soils. Neither chemical nor organic fertilisation is necessary. It can be cultivated without having to modify bad drainage or liming the soil to increase pH, therefore, it is a suitable alternative for small-scale herb cultivation on nutrient-poor, waterlogged soils.

## 4. Conclusion

*Utricularia* is an important component of nutrient-poor waterlogged habitats for which its special body plan and carnivorous habit are adapted. Being sensitive to microhydrological changes, water trophicity, biotic, and chemical pollutions, the presence or absence of common or rare *Utricularia* species could serve as indicator to predict the general health and recovery of many wet microhabitat types. The versatility of common species, on the other hand, gives them an edge over other medicinal herbs on acid, wet poor soils usually deemed not suitable for any crops.

## Figures and Tables

**Table 1:** General habit of Peninsular Malaysian *Utricularia* found in mountainous sites.

Species	General habit	Leaf	Flower	Fruit
*U*.* aurea *	Free floating	Much divided	Yellow	Pendent
*U*.* bifida *	Terrestrial/semiaquatic	Filiform	Yellow	Enclosed by calyx
*U*.* caerulea *	Terrestrial/semiaquatic	Narrow obovate	Pink	Crowded near tip
*U*.* furcellata *	Lithophytic/terrestrial	Rosette, spatulate	Pink/white	Calyx bonnet-like
*U*.* gibba *	Free floating	Divided, filiform	Yellow	Minute, globular
*U*.* involvens *	Terrestrial/semiaquatic	Ribbon-like	Yellow, twines	Enclosed by calyx
*U*.* minutissima *	Terrestrial/semiaquatic	Filiform	Purple/white	Minute, ellipsoid
*U*.* scandens *	Terrestrial	Filiform	Yellow, twines	Enclosed by calyx
*U*.* striatula *	Lithophytic/epiphytic	Rosette, spatulate	Pink/white	Calyx bonnet-like
*U*.* uliginosa *	Terrestrial/semiaquatic	Ribbon-like	Bluish	Enclosed by calyx
*U*.* vitellina *	Terrestrial, on moss	Ribbon-like	Yellow	Enclosed by calyx

**Table 2:** Peninsular Malaysian *Utricularia* specimens from mountainous areas examined.

Species	Collector, specimen number (Herbarium of deposit)
*U*.* aurea *	Burkill, HMB2327 (L, SING); Purseglove, P4293 (K, L, SING)

*U*.* bifida *	Chew, FRI53756 (K, KEP); Chew, FRI67377 (DUB, K, KEP); Kiew, RK2232 (KEP); Siti-Munirah, FRI55252 (KEP)

*U*.* caerulea *	Burkill, HMB 3305 (K, L, SING); Chew, FRI63329 (DUB, K, KEP, TAIF); Ridley, 10091 (SING); Ridley, *s*.*n*. (SING); Robinson, 6112 (K, SING); Spare, 2925 (SING); Spare, 3667 (SING); Wight, 2418 (L)

*U*.* furcellata *	Chew, FRI53603 (KEP)

*U*.* gibba *	Spare, 3615 (SING)

*U*.* involvens *	Abdul Kadir SF19763 (L, SING); Burkill, HMB 3306 (K, SING); Chew FRI63280 (KEP, K, DUB, TAIF); Ding, 783 (K, L); Flippance, *s*.*n*. (SING); Kiew, RK4859 (SING); Mohd. Haniff 5174 (SING); Mohd. Haniff, SF4736 (L, SING); Ng, FRI27117 (KEP); Reilly, 155 (K); Ridley, *s*.*n*. (SING); Robinson, HCR 5959 (K, SING); Symington, FMS46881 (KEP); Anonymous, SFN35814 (SING)

*U*.* minutissima *	Chew, FRI60205 (KEP); Chew, FRI63300 (K, KEP); Chew, FRI63328 (DUB, KEP); Chew, FRI65651 (KEP); Chew, FRI67378 (DUB, K, KEP); Hislop, *s*.*n*. (SING); Holttum, 20645 (SING); Kiew, RK2438 (SING); Kloss, 12132 (SING); Kloss, 12206 (L, SING); Mohd. Haniff, 7994 (K, L, SING); Ridley, 16111 (K, SING); Ridley, 16112 (K, SING); Ridley, s.n. (L); Ridley, *s*.*n*. (SING); Robinson, 5955 (K, SING); Spare 3664 (K, L, SING); Spare, 3665 (SING); Symington, FMS37774 (KEP); Symington, 28840 (SING); Kiew, RK4080 (K, KEP); Wilkie, FRI52872 (E, KEP); Wong, W171 (KEP); Wray, 5447 (L, SING);

*U*.* scandens *	Chew, FRI63327 (KEP, K, DUB); Ridley, *s*.*n*. (L, SING)

*U*.* striatula *	Burkill, HMB3362 (K, L); Chew, FRI58683 (KEP, SING); Chew, FRI58685 (KEP); Chew, FRI60221 (KEP); Chew, FRI65653 (KEP); Chew, FRI67363 (KEP, K); Chew, *s*.*n*. (KEP); Curtis, *s*.*n*. (SING); Kiew, RK2434 (KEP); Kloss, 12131 (SING); Kloss, 12214 (SING); Lim, FRI56349 (E, KEP, L, SAN, SING); Ridley, *s*.*n*. (SING); Robinson, 5970 (K); Robinson, 5976 (SING); Spare, 3663 (SING); Stone, 6394 (L); Wight, 2419 (L); Holttum, 21595 (SING); Kiew, RK230 (SING); Mohd. Haniff, 7883 (SING); Ridley, 16111 (K, SING); Seimund, ES170 (SING); Wilkie, FRI52874 (E, KEP, SAN); Wong, W92 (KEP); Wray, 3880 (SING); Wray, 4146 (SING); Yapp, 436 (K)

*U*.* uliginosa *	Chew, FRI58682 (KEP, SING); Chew, FRI58684 (KEP); Chew, FRI 63326 (DUB, K, KEP, TAIF); Chew, FRI 63285 (DUB, K, KEP, SING); Hislop, *s*.*n*. (L, SING); Rao, 90 (L); Ridley, *s*.*n*. (SING); Spare, 3662 (SING); Symington, FMS37773 (KEP)

*U*.* vitellina *	Chew, FRI60222 (KEP); Chew, FRI63684 (KEP); Ridley 16113 (SING)

Herbaria code: DUB = Dublin, K = Kew, KEP = Kepong, L = Leiden, SING = Singapore, TAIF = Taipei.

**Table 3:** Commonness criteria* for *Utricularia* of Peninsular Malaysia.

Category	Distribution in Peninsular Malaysia	Disturbance adaptability	Collection frequency^f^
Common	Throughout	Common in man-made site	>40
Fairly common	Throughout	Mostly in natural site	20–40
Narrow range	<3 localities	Mostly in rarely disturbed site	10–20
Rare	<2 localities	Only in pristine site	<5

^f^based on number of herbaria specimens.

*adapted to suit the geographical range and demographic details on population of the Taxon Data Information Sheets modified from the IUCN Red list assessment questionnaire, as recommended by the Malaysia Plant Red List guidebook [9].

**Table 4:** *Utricularia* of Peninsular Malaysia montane microhabitat details.

Species	Montane microhabitat type	Altitude (m a.s.l.)	pH range
Common and fairly common species			

*U*.* aurea *	Reservoir or man-made ponds	0–1,231	3–7
*U*.* bifida *	Wayside puddles, damp sandy spot	4–1,190	4–6
*U*.* gibba *	Reservoir or ponds	0–1,577	3–5.5
*U*.* minutissima *	Heaths, stream banks, damp spots, puddles	1–2,180	4–6
*U*.* striatula * ^∆^	Wet/dripping rock faces/tree trunks/branches, mossy mounds	150–2,180	3.5–5.5
*U*.* uliginosa *	Stream beds, *Sphagnum* bogs	1–1,362	3.5–6.5

Narrow Range Species			

*U*.* caerulea *	Stream banks	1–901	4.5–6
*U*.* involvens * ^∆^	Waterfalls, damp grassy spots	750–1,189	3.5–6

Rare Species			

*U*.* furcellata * ^∆^	Heaths	1,500	*c*. 5
*U*.* scandens * ^∆^	Stony heaths, stream banks, damp grassy spots	380–387	*c*. 5
*U*.* vitellina * ^∆^	Stream banks, mountain tops well-aerated damp mossy mounds	1,526–2,080	3.5–5

m a.s.l. = metre above sea level.

^∆^Essentially mountain species in hilly microhabitats above 300 m altitude.
